# Antioxidants and phytochemicals – a scoping review for Nordic Nutrition Recommendations 2023

**DOI:** 10.29219/fnr.v67.10324

**Published:** 2023-12-01

**Authors:** Mari C. W. Myhrstad, Alicja Wolk

**Affiliations:** 1 Department of Nursing and Health Promotion, Faculty of Health Sciences, Oslo Metropolitan University, Oslo, Norway; 2 Institute of Environmental Medicine, Karolinska Institutet, Stockholm, Sweden

**Keywords:** antioxidants, phytochemicals, phenols, oxidative stress, resveratrol, nutrition recommendations

## Abstract

Antioxidants are a collection of substances that may prevent or delay the oxidation of cellular components. The antioxidant defense system includes both endogenously produced antioxidants and dietary antioxidants. The consumption of dietary antioxidants has long been speculated to be important for the defense against cellular oxidation, inflammation, and other disease-related processes. In addition to the well-known dietary antioxidants, such as vitamin C, vitamin E, β-carotene, and selenium, whole plants and plant-products contain numerous compounds, called phytochemicals, with antioxidant properties. These phytochemicals are potentially important modulators of oxidative stress and have been linked to health beneficial effects. However, the mechanisms underlying these potential health beneficial effects are not well understood. Foods containing high levels of phytochemicals with antioxidant properties include berries, fruits, vegetables, whole grains, and nuts and seeds. The aim of this scoping review is to describe the evidence of the role of specific antioxidants and phytochemicals, but not foods rich in these substances, for health outcomes. Based on a literature search from 2011 to March 2022, we identified eight meta-analyses related to the current topic. These studies include evidence of the effect of resveratrol (present mainly in berries, grapes, and peanuts) on health outcomes related to cardiometabolic risk, blood pressure, obesity, oxidative stress, adipokines, inflammation, and bone quality. In summary, resveratrol did elicit several health beneficial effects. However, the magnitude of effects was low, and whether the effects are related to the redox properties of resveratrol is not known. Even though there is a large body of evidence linking a plant-based diet rich in antioxidants and phytochemicals to beneficial health effects, the role of specific antioxidants and phytochemicals is still unclear.

## Popular scientific summary

- Antioxidants are substances in foods and in the body that may prevent or delay the oxidation of cellular components
- Phytochemicals are secondary plant metabolites with antioxidant properties, of which phenols comprise the largest and most studied group
- Oxidative stress is implicated in several chronic diseases, including cardiovascular disease, cancer, diabetes, neurodegenerative disease, lung disease, and kidney disease
- Total antioxidant capacity in plasma is considered a valid biomarker of dietary antioxidant intake
- Foods with high levels of antioxidants and phytochemicals include berries, fruits, vegetables, whole grains, nuts and seeds, and beverages, such as tea and coffee
- The causal relationship between specific antioxidants and phytochemicals and health outcomes is still unclear, and high doses from dietary supplements may cause adverse effects

The human body is constantly exposed to oxidative processes *via* free radicals and other reactive oxygen and nitrogen species (ROS and RNS) that are formed endogenously under normal cellular metabolic reactions (1). Such reactive species may also develop as a result of disease or exposure to tobacco smoke, environmental pollutants, drugs, radiation, excessive alcohol consumption, and other unknown factors. They have an important role in cellular signaling and in our defense against microorganisms and are therefore required to maintain homeostasis. However, in excess, these reactive substances can react with and potentially alter the structure and function of cellular components, such as lipids, proteins, carbohydrates, RNA, and DNA (2). Antioxidants are a collection of substances that may prevent or delay the oxidation of cellular components (3). The antioxidant defense system includes both endogenously produced antioxidants and antioxidants from exogenous sources (dietary antioxidants). Oxidative stress is the condition where oxidative damage is accumulated in the body due to an imbalance between the endogenous generation of ROS or RNS and the elimination by the antioxidant defense system (2, 4, 5). Oxidative stress is considered to play a role in the pathogenesis of aging and degenerative diseases (5). The consumption of dietary antioxidants may therefore be an important factor for the defense system of the body against oxidation, inflammation, and other disease-related processes.

In addition to the well-known dietary antioxidants, such as vitamin C, vitamin E, β-carotene, and selenium, whole plants and plant extracts contain numerous known and unidentified compounds, called phytochemicals, with antioxidant properties (1). Phytochemicals are potentially important modulators of oxidative stress and have been linked to beneficial health effects. However, the mechanisms underlying these potential health beneficial effects are not well understood. Whether or not they exert beneficial effects due to their ability to act as antioxidants, or through other mechanisms, is not always clear. Foods containing high levels of antioxidants and phytochemicals with antioxidant properties include several berries (blueberries, blackberries, strawberries, raspberries, etc.), fruits (pomegranates, grapes, oranges, lemons, etc.), whole grains, nuts and seeds (walnuts, sunflower seeds, etc.), vegetables (kale, red cabbage, legumes, pepper, etc.), and drinks (green/black tea, coffee, cacao, red wine, etc.) (6).

The Nordic Nutrition Recommendations (NNR) from 2012 provided no recommendations for specific phytochemicals and antioxidant-rich foods, other than the general dietary recommendation of 500 gram per day of fruits and vegetables (7). Intake of antioxidants in the form of dietary supplements, either individually or in combination, was discouraged. This scoping review summarizes the updated evidence accumulated from 2012 to the current date and includes evidence related to health outcomes for antioxidants and phytochemicals that are not covered in other scoping reviews of the NNR2023 project (8). Plant-based products rich in antioxidants and phytochemicals, such as tea, coffee, cacao, fruit, and vegetables, and dietary antioxidants, such as vitamin C, vitamin E, β-carotene, lycopene, selenium, and zinc, are therefore not included in the present review and will not be the focus of the text. The aim is to describe the evidence of the role of specific antioxidants and phytochemicals, but not foods rich in these substances, for health outcomes of relevance for NNR2023 (Box 1).

Box 1 Background papers for Nordic Nutrition Recommendations 2023

- This paper is one of many scoping reviews commissioned as part of the Nordic Nutrition Recommendations 2023 (NNR2023) project (8)
- The papers are included in the extended NNR2023 report, but, for transparency, these scoping reviews are also published in Food & Nutrition Research
- The scoping reviews have been peer reviewed by independent experts in the research field according to the standard procedures of the journal
- The scoping reviews have also been subjected to public consultations (see report to be published by the NNR2023 project)
- The NNR2023 committee has served as the editorial board
- While these papers are a main fundament, the NNR2023 committee has the sole responsibility for setting dietary reference values in the NNR2023 project

## Methods

This scoping review follows the protocol developed within the NNR2023 project (8). The sources of evidence used follow the eligibility criteria described previously (9) and include evidence for the intake of antioxidants and phytochemicals and related health outcomes that are not covered in other parts of NNR2023. Accumulated evidence about health outcomes related to the consumption of plant-based products rich in antioxidants and phytochemicals, such as tea, coffee, cacao, fruit, vegetables, and red wine, and well-known dietary antioxidants, such as vitamin C, vitamin E, β-carotene, lycopene, selenium, and zinc, was therefore excluded. No qualified systematic review (qSR) within the scope of this scoping review was identified from the paper by Høyer et al. (10). Furthermore, no *de novo* systematic review on the current topic was prioritized by the NNR2023 project.

**Fig. 1:**
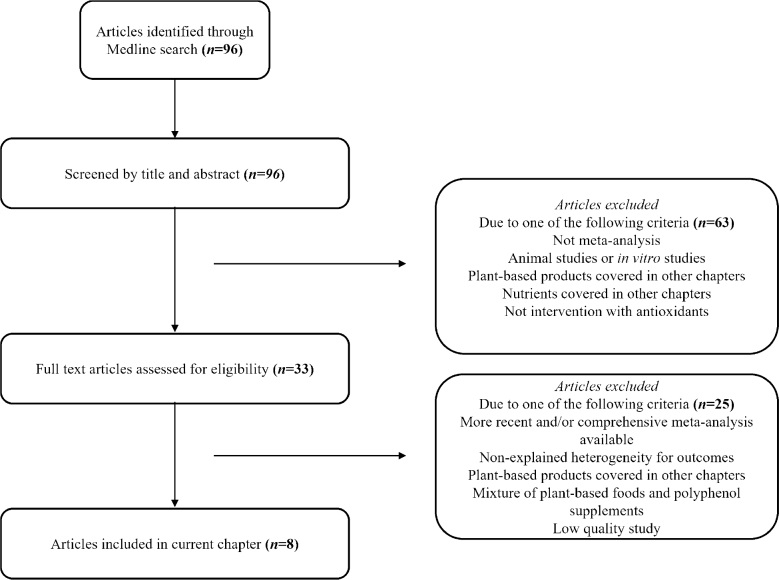
The flow chart of the article selection.

A literature search was performed in MEDLINE (last performed 16th of March 2022) to summarize the evidence related to the intake of antioxidants and phytochemicals and health outcomes published from 2011 to current date. An updated search was performed on the 1st of February 2023. The search string for the MEDLINE search was *(exp Antioxidants/ AND exp Phytochemicals/ OR exp Polyphenols/ limited to English language and humans and yr = ‘2011 –Current’ and systematic review).* The search resulted in altogether 96 publications (Fig. 1). Titles and abstracts were reviewed for relevance, and we included 33 articles that clearly or possibly fulfilled the following criteria: meta-analysis, human studies, antioxidants, and phytochemicals that were not covered in other parts of NNR2023. Altogether, 63 articles were excluded due to the following reasons: not meta-analysis, animal or *in vitro* studies, intervention with plant/food products and/or nutrients covered in other NNR2023 reviews, and/or not intervention with antioxidants. Full texts of the 33 included articles were thereafter assessed for eligibility. Of the 33 articles, 25 articles were excluded due to the following reasons: more recent and/or comprehensive meta-analysis available, non-explained heterogeneity for outcomes, mixture of foods rich in polyphenols and polyphenol supplements, and low-quality study. In total, eight articles were identified as eligible and reviewed in this current scoping review (Table 1). Figure 1 shows the flow chart of the study selection. The list of articles excluded after reading full text, and the reason for exclusion is presented in Supplementary Table 1.

**Table 1:** Table of included meta-analyses for summarizing possible health effects linked to the intake of antioxidant and phytochemicals. The meta-analyses are reported based on outcome measures as given by the authors.

Study	Study design (number of studies)	Intervention	Population characteristics	Outcome measures	Results given as SMD/WMD (95% CI)^a^	Evidence for heterogeneity^b^	Comments
* Zeraattalab-Motlagh et al. (2021), Am J Clin Nutr (43)	RCT (*n* = 28 RCT from 11 meta-analysis)	Resveratrol (8–3,000 mg/d) Duration: 4–48 w	1,476 participants with T2D (ranging from 77 to 789 for reported variables)	***T2D*** 29 variables, 9 variables with moderate-certainty evidence are reported	Reduction in: **SBP –**1.23 (**–**1.96, **–**0.49), **DBP –**0.85 (**–**1.56, **–**0.13), **MAP –**4.21 (**–**7.35, **–**1.07), **PP –**6.52 (**–**11.20, **–**1.84), **HOMA-IR –**0.46 (**–**0.74, **–**0.11), **FGC –**0.33 (**–**0.57, **–**0.09) Increase in: **GGT** 2.01 (0.70, 3.32, **AST** 1.23 (0.29, 2.16), **adiponectin** 1.37 (0.15, 2.60) No effect: ALT, TG, BMI, and WC	Significant: SBP, DBP	Only results related to T2D are given. Certainty of evidence (GRADE) for the other studied variables (9 of totally 29) was evaluated as low.
* Zeraattalab-Motlagh et al. (2021), Am J Clin Nutr (43)	RCT (*n* = 15 RCT from 9 meta-analyses)	Resveratrol (100–3,000 mg/d) Duration: 4–24 w	727 participants with MetS (407 participants for the reported variable)	***MetS*** 24 variables, 1 variable with moderate-certainty evidence is reported	No effect: HDL	Not significant for reported variable	Only results related to MetS are given. Certainty of evidence (GRADE) for the other studied variables (23 of totally 24) was evaluated as low.
* Zeraattalab-Motlagh et al. (2021), Am J Clin Nutr (43)	RCT (*n* = 6 RCT from 10 meta-analyses)	Resveratrol (150–3,000 mg/d) Duration: 8-24 w	271 participants with NAFLD (ranging from 156 to 216 for reported variables)	***NAFLD*** 24 variables, 6 variables with moderate-certainty evidence are reported	Reduction in: **BW** –0.67 (–1.26, -0.08), **BMI** –0.25 (–0.45, -0.04), **DBP** –0.40 (–0.79, -0.02). No effect: TG, MAP, or WC	Not significant for reported variables	Only results related to NAFLD are given. Certainty of evidence (GRADE) for the other studied variables (18 of totally 24) was evaluated as low.
***Mousavi et al.*** (2019), Obesity Reviews (44)	RCT (*n* = 28)	Resveratrol (8–3,000 mg/d) Duration: 4–52 w	1,514 subjects with BMI in the range from 23 to 35.1 kg/m^2^	***Obesity*** 4 variables	Reduction in: **BW** –0.51 (-0.94, -0.09), **BMI** –0.17 (-0.32, –0.03), **WC** –0.79 (–1.39, -0.2) No effect: FM	Significant: BW	Subgroup analysis: Reduction in BW and BMI in trials with doses <500 mg/d, duration >3 months and performed on participants with obesity
***Akbari et al.*** (2019), High Blood Pressure & Cardiovascular Prevention (45)	RCT (*n* = 28)	Resveratrol (40–3,000 mg/d) Duration: 1–48 w	1,748 participants with MetS or related disorders	***BP*** 3 variables	Increase in: **FMD** 1.77 (0.25, 3.29) No effect: SBP or DBP	Significant: FMD, SBP, and DBP due to duration and type of disease	Subgroup analyses: SBP and DBP decreased in trials with T2D compared to other diseases
***Gorabi et al.*** (2021), Phytotherapy Research (46)	RCT (*n* = 35)	Resveratrol (8–3,000 mg/d) Duration: 4–48 w	1,128 participants with MetS, CVD, CAD, stable angina, overweight, T2D, older, postmenopausal women, hypertension, RA, or PCOS	***Inflammation*** 2 variables	Reduction in: **hs-CRP** –0.40 (–0.70 to –0.09), **CRP** -0.47 (–0.69, –0.25)	Significant: hs-CRP	Subgroup analysis: hs-CRP and CRP decreased in trials of longer duration (≥10 w) and with doses ≥500 mg/d (CRP)
***Koushki et al.*** (2018), Clinical Therapeutics (47)	RCT (*n* = 17)	Resveratrol (6–800 mg/d) Duration: 4–52 w	736 participants with CVD, T2D, NAFLD, healthy normal weighted, obese, or angina pectoris	***Inflammation*** 3 variables	Reduction in: **TNFα** –0.44 (–0.71, –0.164), **hs-CRP** –0.27 (–0.5, –0.02) No effect: IL-6	Significant: hs-CRP, IL-6, and TNF**α**.	Significant heterogeneity was observed for the type of sample in IL-6 and study duration for IL-6, TNF-**α**, and hs-CRP.
***Mohammadi-Sartang et al.*** (2017), Pharmacological Research (49)	RCT (*n* = 9)	Resveratrol (16–3,000 mg/d) Duration: 4–48 w	590 participants with obesity, T2D, NAFLD, CVD, postmenopausal women, or healthy elderly	***Adipokines*** 2 variables	Increase in: **Adiponectin** 1.10 (0.88, 1.33) No effect: Leptin	Not significant for reported variables	Subgroup analysis: Greater adiponectin-reducing effect in trials with doses >100 mg/d Compared to doses <100 mg/d
***Koushki et al.*** (2020), Postgraduate Medical Journal (48)	RCT (*n* = 12)	Resveratrol (40–3,000 mg/d) Duration: 4–26 w	575 participants with T2D, healthy normal weighted or obese, NAFLD, chronic kidney disease, nephropathy, hypercholesterolemic or ulcerative colitis	***Oxidative stress*** 4 variables	Increase in: **TAC** 0.52 (–0.02, 1.07) No effect: SOD, CAT, and GPx	Significant: TAC, SOD, CAT, and GPx	Subgroup analysis: Resveratrol in doses between 500 and 800 mg/d and treatment >60 d changed the oxidative stress markers
***Li et al.*** (2021), BMC Complementary Medicine and Therapies (50)	RCT (*n* = 10)	Resveratrol (8–1,500 mg/d) Duration: 4–52 w	698 participants with T2D, NAFLD, obesity, healthy elderly, or postmenopausal women	***Bone quality*** 9 variables	No effect: BMD, hip BMD, whole body BMD, bone serum markers (ALP, BAP, OCN, PINP, CTX, and PTH)	Not significant for reported variables	Subgroup analysis: BMD and serum bone markers were not affected by dose, intervention duration, and pathology of participants

a Significant results are given as standardized mean differences (SMD) or weighted mean differences (WMD) with 95% CI compared to placebo and are indicated in bold.

b Significant heterogeneity if *I*^2^ > 50% or *p* > 0.05.

SMD: standardized mean differences; RCT: randomized controlled trials; BP: blood pressure; SBP: systolic BP; DBP: diastolic BP; FMD: flow-mediated dilatation; MAP: mean arterial pressure; TC: total cholesterol; TG: trigycerides; HDL: high density lipoprotein; LDL: low density lipoprotein; Apo-A: apoplipoprotein A; Apo-B: apoplipoprotein B; HOMA-IR: homeostatic model assessment-insulin resistance; FGC: Fasting glucose concentration; ALT: aminotransferase; ASDT: aspartate aminotransferase; GGT: γ-glutamyl transferase; ALP: alkaline phosphatase; IL-6: interleukin-6; NAFLD: non-alcoholic fatty liver disease; BMD: bone mineral density; PCOS: polycystic ovary syndrome; BW: body weight; BMI: body mass index; WC: weight circumferences; T2D: Type 2 diabetes mellitus; MetS: Metabolic syndrome; NAFLD: non-alcoholic fatty liver disease.

* This study reported outcomes related to T2D, MetS, and NAFLD.

## Physiology

### Chemical aspects of phytochemicals

Phytochemicals are considered secondary plant metabolites and are classified according to their chemical structure and functional characteristics. In the plant, they have important functions linked to protection against pathogens and UV radiations in addition to providing color and flavors to the plants (11). The main groups of phytochemicals are phenols, carotenoids, S-containing compounds, and alkaloids, where the phenols comprise the largest and most studied group of phytochemicals with more than 8,000 structural variants (12). The phenols are further divided into classes depending on their chemical structure. These classes include phenolic acids, flavonoids, stilbenes, and lignans (13). Resveratrol is one of the most well-known stilbenes (3,5,4’-trihydroxystilbene) and is produced by several plants, such as berries, grapes, and peanuts (14). During recent years, resveratrol has attained a lot of attention due to its potential health beneficial effects. This is also reflected in this review since all the included meta-analyses from the search in MEDLINE were related to health effects of resveratrol supplementation.

### Metabolism of phytochemicals

Due to the wide variety of chemical structures, the precise mechanisms related to absorption and metabolism of phytochemicals are not well understood. It is, however, known that bioavailability of phytochemicals depends on factors, such as chemical structure, deconjugation in the intestine, absorption, and enzymes available for metabolism (14). In the plants, phytochemicals are generally conjugated to glycosides, or they are sulfated. In addition, they may also appear in a non-conjugated form (aglycons). In general, the conjugated forms need to be hydrolyzed by hydrolases in the intestinal brush border or by enzymes produced by the gut microbiota, whereas the aglycons can be taken up directly. The deconjugation can also start by the chewing process in the mouth and in the stomach. The main site for metabolism of phytochemicals is in the liver, but other tissues such as kidneys and intestinal mucosa have the ability to metabolize phytochemicals. In the liver, phytochemicals are metabolized by phase I and phase II enzymes to yield more soluble compounds and conjugated by glucuronidation, sulfation, methylation, and acetylation (15). Due to their extensive conversion into other metabolites, the bioavailability of most phytochemicals, including the main groups of polyphenols, is considered low. Whether the converted metabolites represent bioactive compounds is not always known. Furthermore, several enzymes involved in metabolization of phytochemicals, and in particular the phase I and II enzymes, show high genetic variability due to single nucleotide polymorphisms (SNPs) (15). It has been speculated that the different genetic variants may affect the bioavailability and can thus give individual differences in the benefits obtained from the consumption of dietary phytochemicals (15). The gut microbiota will also influence the bioavailability of phytochemicals through their ability to produce enzymes that can metabolize and deconjugate the phytochemicals and thereby facilitate intestinal absorption (16). In addition, phytochemicals may modulate the gut microbiota and change the composition toward beneficial bacteria that can produce metabolites such as short chain fatty acids (SCFAs) (17). SCFAs have been shown to impact host metabolic processes (18). However, the exact role of gut microbiota in the bioavailability and function of phytochemicals is not known.

Regarding resveratrol, metabolization in the intestine and liver produces metabolites with lower biological activity than the parent resveratrol. Resveratrol may also be converted from the active *trans* to the less active *cis* form by oxidative enzymes in the liver (19). In addition, the low water solubility of resveratrol will also affect the compound’s absorption and bioavailability (19). Even so, resveratrol has been linked to health beneficial effects, indicating that that the compound is bioactive even in low concentrations (20).

### Molecular functions of phytochemicals

Phytochemicals have been shown to possess a wide variety of biological functions, including antioxidant, anti-apoptosis, anticarcinogen, anti-inflammation, and anti-atherosclerosis properties, improvement of the endothelial function, as well as inhibition of angiogenesis (21, 22). Most of these biological functions have been linked to their antioxidant properties by the virtue of redox reactions (2). Phytochemicals in plant foods contribute to two important components of the antioxidant defense system: 1) the ability to scavenge or neutralize free radicals directly and 2) the ability to induce endogenous antioxidants. It is well known that phytochemicals present in food may induce endogenous antioxidants through direct activation of transcription factors or by the activation of cell signaling, leading to the expression of antioxidant genes that are important in maintenance of metabolic homeostasis or cell integrity (23).

Redox reactions involve both oxidation and reduction and occur simultaneously. The redox-active compound can be an antioxidant in one system (such as a plant subcellular system or an *in vitro* system) but inactive or even a prooxidant in another biological system (24). Given this notion, redox-active phytochemicals in whole food may form complexes or work synergistically to exert an enhanced effect. Therefore, the observed health benefits of a plant-based diet rich in antioxidants and phytochemicals are not comparable to the effect of dietary supplements alone (2). Antioxidants and phytochemicals may also provide health benefits through other mechanisms not related to their redox properties (25). In this regard, resveratrol is a known activator of Sirtuin (SIRT-1). SIRT-1 possesses deacetylation activity and is involved in metabolic processes, including glycemic regulation and lipid metabolism (26). Resveratrol is also known to have anti-inflammatory properties through the activation of inflammatory signaling pathways (27).

## Assessment of antioxidant status

The total antioxidant capacity (TAC) concept describing an antioxidant’s capacity for reducing oxidants provides an assessment of antioxidant activity and synergistic interactions of redox molecules (28). TAC can be applied to both biological systems and foods, and it is defined as the moles of radicals neutralized per liter (or gram) of tested sample (29). TAC in plasma (fasting) can be assessed with different laboratory methods: by oxygen radical absorbance capacity (ORAC), total radical-trapping antioxidant parameters (TRAP), and ferric-reducing antioxidant power (FRAP) (28). Markers of antioxidant status and oxidative stress can be measured in plasma, spot urine, and saliva using spectrophotometric and fluorometric methods (30). Polyphenols can be measured in urine and plasma. Spot urinary polyphenols have potential as a biomarker of polyphenol-rich food intakes. Findings suggest that total urinary polyphenols may be a promising biomarker of total polyphenol intakes from foods and drinks, and that hippuric acid specifically may be a biomarker of total polyphenol intakes and polyphenols from tea/coffee (31). The literature highlights the utility of hippuric acid in urine and plasma as an indicator of low fruit and vegetable intake and changes in gut microflora (32).

In a group of middle-age and elderly Swedish women (mean age 62 years, mean BMI 25.6 kg/m^2^), the plasma TAC was analyzed with the ORAC assay described by Prior et al. (33). The measured values ranged from 6,092 to 16,967 μmol/L, and the mean value (SD) of ORAC total was 11,194 (2,504) μmol/L (34).

## Dietary intake in Nordic and Baltic countries

There are few studies from Nordic countries that present estimates of dietary TAC (dTAC) intake based on food-frequency questionnaires (FFQ). Such estimates of dTAC reflect the sum of dietary antioxidant intake from single foods and take into account synergistic effects between different antioxidants within each food. It was shown that dTAC values correlated with plasma TAC status and represent valid and reproducible estimates that may be used in nutritional epidemiology to assess antioxidant intake from foods (34). In a study of elderly Swedish men (mean age 61 years, 25.8 kg/m^2^) and women (62 years, BMI 25.0 kg/m^2^), estimates of total daily intake of dTAC based on the sum of antioxidant capacity of individual foods as assessed with ORAC assay were 14,025 (median) μmol Trolox equivalents/day in men and 12,353 (median) in women (35). Fruits and vegetables were the major contributors to FFQ-based dTAC-ORAC estimates. Food contributors to FFQ-based estimates of dTAC in women were as follows: fruits and vegetables 56.5%, grain products 19.7%, tea 9.5%, chocolate 4.9%, juice 3.9%, and wine 2.5% (34). In a study of Swedish children, the mean (SD) total daily intake of antioxidants at the age of 8 years, as estimated by FFQ-based dTAC-ORAC, was 10,397 (4,322) μmol Trolox equivalents/day for girls and slightly lower for boys 9,611 (4,486) (36).

## Health outcomes relevant for Nordic and Baltic countries

Oxidative stress has been implicated in several chronic diseases, including cardiovascular disease, cancer, diabetes, neurodegenerative disease, lung disease, and kidney disease due to accumulation of oxidative damage (5, 37, 38). Markers of oxidative damage include a wide variety of compounds, such as enzymes, oxidative DNA adducts, and lipid peroxidation products (2). Dietary antioxidants and phytochemicals may potentially reduce the harmful effects of oxidants. However, the evidence-linking beneficial health effects to their ability to reduce oxidative stress are still scarce. This may be explained by the difficulties to reliable measure oxidative stress *in vivo* because of the transient- and complex-free radical reactions (2). In addition, antioxidants and phytochemicals may provide health benefits through other mechanisms not related to their redox properties (25, 39).

Furthermore, the suggested role of antioxidants and phytochemicals in protection against oxidative damage is often based on *in vitro* studies with high doses and cannot be directly extrapolated to humans. There is also evidence, suggesting that elevated intake of supplements with antioxidant properties (β-carotene, retinol, and tocopherol) increases the risk of total mortality (40, 41). This was recently thoroughly discussed by the US Preventive Services Task Force, and antioxidant supplements are, therefore, not recommended for the general population (42).

To summarize the evidence related to the intake of specific phytochemicals and antioxidants and health outcomes published since 2011, we performed a search as described in the Methods section. A total of eight articles were found to be eligible. These articles include evidence of the effect of resveratrol on health outcomes related to cardiometabolic risk, blood pressure, obesity, oxidative stress, adipokines, inflammation, and bone quality (Table 1). All the included articles are meta-analyses of randomized controlled trials (RCTs), where doses of resveratrol ranged from 6 to 3,000 mg/d, with a duration of trial ranging from 1 to 52 weeks. The articles included both healthy people and people with various metabolic and/or inflammatory diseases.

### Effect of resveratrol supplementation on markers of cardiometabolic risk and obesity

Meta-analyses on the effect of resveratrol supplementation on cardiometabolic risk factors in people with type 2 diabetes (T2D), metabolic syndrome (MetS), and non-alcoholic fatty liver disease (NAFLD) were recently performed by Zeraattalab-Motlagh et al. (43). According to their findings, there was moderate-certainty evidence that resveratrol beneficially changed risk factors related to blood pressure, glycemic regulation, liver enzymes, and adiponectin in people with T2D, and body weight in people with NAFLD, whereas no effect on the reported cardiometabolic risk factors was evident in people with MetS. The authors concluded that the current evidence does not support supplementation of resveratrol for the management of cardiometabolic risk due to the low magnitude of effect (43). In a meta-analysis performed by Mousavi et al., a significant beneficial effect related to body weight was found in people with obesity. The effect was dependent on dose and duration of supplementation (44). Furthermore, Akbari et al. found a significant effect on endothelial function, but not blood pressure, in a meta-analysis performed in people with MetS or related disorders (45).

### Effect of resveratrol supplementation markers of inflammation, oxidative stress, and adipokines

Inflammatory markers, such as TNFα, hsCRP, and CRP, were significantly reduced after interventions with resveratrol in two meta-analyses where both people with various inflammatory diseases and healthy people were included (46, 47). However, due to substantial heterogeneity between the RCTs in both publications and other limitations such as different sources of resveratrol used, the authors concluded that more studies are needed to establish the beneficial effect of resveratrol in prevention of inflammation. In a meta-analysis performed by Kouskhi et al., the effect of resveratrol supplements on makers of oxidative stress was evaluated (48). Even though the circulating TAC significantly increased, no effect of resveratrol supplementation on established markers of oxidative stress was found. The authors discussed the heterogeneity among the studies and concluded that more studies are needed to confirm the results (48). Furthermore, resveratrol supplements significantly increased adiponectin, whereas no effect was found on leptin levels in a meta-analysis (49). The authors also concluded that more studies are needed to confirm the results as the findings were sensitive to one study (49).

### Effect of resveratrol supplementation on bone quality

Supplementation of resveratrol did not significantly change bone serum markers in a meta-analysis, including people with different pathophysiology conditions. The authors discussed that the certainty of the evidence is low (50).

## Requirement and recommended intakes

In the current scoping review, we have summarized the evidence related to specific antioxidants and phytochemicals and health outcomes. As described previously, only meta-analyses of RCTs with resveratrol were identified. The results indicate that supplements with resveratrol may elicit beneficial effects on markers related to inflammation, body weight, blood pressure, adipokines, and glycemic regulation in people with metabolic and/or inflammatory diseases, whereas no effect was found on markers related to lipid metabolism, bone quality, or oxidative stress markers. However, the magnitudes of effects for most variables were low, and whether the effects are related to the redox properties of resveratrol remains to be elucidated. More research on the health effects of antioxidants in general and resveratrol in particular are therefore warranted.

There is a large body of evidence that a plant-based diet rich in antioxidants and phytochemicals reduces the risk of several diseases associated with oxidative stress. In the EPIC cohort study, an inverse relation between the intake of polyphenols and cancer risk has been suggested (51). Furthermore, the intake of specific flavonoids was associated with a lower risk of T2D in the Nurses’ Health Study I and II (52). The evidence linking this protective effect to antioxidant mechanisms is, however, insufficient. This is due to the lack of studies showing effects of individual antioxidants or phytochemicals on oxidative stress markers and markers of antioxidant status. High doses of certain antioxidants or phytochemicals may also be harmful and elicit adverse health effects (42). We still need more scientific evidence to understand the causal relations between dietary antioxidants and phytochemicals and health outcomes. Future research should therefore address the mechanisms underlying the potential health beneficial effects of antioxidants and phytochemicals in human intervention studies.

## Conflict of interest and funding

The authors have not received any funding or benefits from industry or elsewhere to conduct this study.
