# Supplementary material for: Antioxidants and phytochemicals – a scoping review for Nordic Nutrition Recommendations 2023
Source: Food Nutr Res. 2023 Dec 1;67:10.29219/fnr.v67.10324. doi: 10.29219/fnr.v67.10324 (PMC10710867; doi:10.29219/fnr.v67.10324)
Supplement: Supplementary file 1 [file FNR-67-10324-s001.docx]

**Supplementary Table 1.** List of excluded studies

| **Ref details** | **Intervention** | **Outcome measures** | **Reason for exclusion** |
| --- | --- | --- | --- |
| ***Asis et. al.***  (2019)  Ann of the New York Academy of Sciences | Resveratrol | Biomarkers of bone health | More recent and/or more comprehensive meta-analysis available (1) |
| ***Fogacci et. al.***  (2019)  Critical Reviews in Food Science & Nutrition | Resveratrol | Blood pressure | More comprehensive meta-analyses available also covering obese individual (2) |
| ***Marx et. al.***  (2018)  Nutrition reviews | Resveratrol | Cognitive performance | Non-explained heterogeneity for outcomes |
| ***Farzaei et. al.***  (2018)  Pharmacological research | Resveratrol | Cognitive/memory performance | Non-explained heterogeneity for outcomes |
| ***d'Unienville et. al.***  (2021)  Journal of the International Society of Sports Nutrition | Plant-based foods rich in polyphenols | Exercise performance | Plant-based products covered in other chapters |
| ***Somerville et. al.***  (2017)  Sports medicine | Mixture of plant-based products and polyphenol supplements | Exercise performance | Mixture of plant-based foods and polyphenol supplements |
| ***Haghighatdoost et. al.***  (2019)  European Journal of Clinical Nutrition | Resveratrol | Inflammation | More recent and/or more comprehensive meta-analyses available (3, 4) |
| ***Tabrizi et. al.***  (2018)  Food & function | Resveratrol | Inflammation | More recent and/or more comprehensive meta-analyses available (3, 4) |
| ***Sahebkar et. al.***  (2015)  International journal of cardiology | Resveratrol | Inflammation | More recent and/or more comprehensive meta-analyses available (3, 4) |
| ***Kilchoer et. al.***  (2020)  Nutrients | Resveratrol | Lipid profile | Low quality study |
| ***Haghighatdoost et. al.***  (2018)  Pharmacological Research | Resveratrol | Lipid profile | Non-explained heterogeneity for outcomes |
| ***Sahebkar et. al.***  (2013)  Nutrition Reviews | Resveratrol | Lipid profile | More recent and/or more comprehensive meta-analysis available (5) |
| ***Akbari et. al.***  (2020)  Lipids in Health & Disease | Resveratrol | Lipid profile and liver enzymes | More recent and/or more comprehensive meta-analysis available (5) |
| ***Wei et. al.***  (2021)  Complementary therapies in medicine | Resveratrol | Variables related to NAFLD | More recent and/or more comprehensive meta-analyses available (5) |
| ***Jakubczyk et. al.***  (2020)  Nutrients | Resveratrol | Variables related to NAFLD | More recent and/or more comprehensive meta-analyses available (5) |
| ***Elgebaly et. al.***  (2017)  Journal of gastrointestinal and liver diseases | Resveratrol | Variables related to NAFLD | More recent and/or more comprehensive meta-analyses available (5) |
| ***Darand et. al.***  (2021)  International journal of clinical practice | Resveratrol | Variables related to NAFLD | More recent and/or more comprehensive meta-analyses available (5) |
| ***Rafiee et. al.*** (2021), Complementary therapies in clinical practice | Resveratrol | Variables related to NAFLD | More recent and/or more comprehensive meta-analyses available (5) |
| ***Delpino et. al.***  (2021)  Clinical nutrition ESPEN | Resveratrol | Variables related to obesity | Non-explained heterogeneity for outcomes |
| ***Tabrizi et. al.***  (2020)  Critical reviews in food science and nutrition | Resveratrol | Variables related to obesity | Non-explained heterogeneity for outcomes, low quality |
| ***Omidian et. al.***  (2020)  Endocrine, metabolic & immune disorders drug targets | Resveratrol | Variables related to oxidative stress | More recent and/or more comprehensive meta-analyses available (6) |
| ***Hosseini et. al.***  (2020)  Complementary therapies in medicine | Resveratrol | Variables related to T2D | More recent and/or more comprehensive meta-analyses available (5) |
| ***Jeyaraman et. al.***  (2020)  The Cochrane database of systematic reviews | Resveratrol | Variables related to T2D | More recent and/or more comprehensive meta-analyses available (5) |
| ***Zhao et al.***  (2019)  Obesity | Resveratrol | Variables related to T2D | More recent and/or more comprehensive meta-analyses available (5) |
| ***Hausenblas et. al.***  (2015)  Molecular nutrition & food research | Resveratrol | Variables related to T2D | More recent and/or more comprehensive meta-analyses available (5) |

T2D: Type 2 diabetes mellitus, MetS: Metabolic syndrome, NAFLD: non-alcoholic fatty liver disease.

**References:**

1. Li Q, Yang G, Xu H, Tang S, Lee WY. Effects of resveratrol supplementation on bone quality: a systematic review and meta-analysis of randomized controlled trials. BMC Complement Med Ther 2021;21(1):214. doi: 10.1186/s12906-021-03381-4.

2. Akbari M, Tamtaji OR, Lankarani KB, Tabrizi R, Dadgostar E, Kolahdooz F, Jamilian M, Mirzaei H, Asemi Z. The Effects of Resveratrol Supplementation on Endothelial Function and Blood Pressures Among Patients with Metabolic Syndrome and Related Disorders: A Systematic Review and Meta-Analysis of Randomized Controlled Trials. High Blood Press Cardiovasc Prev 2019;26(4):305-19. doi: 10.1007/s40292-019-00324-6.

3. Gorabi AM, Aslani S, Imani D, Razi B, Sathyapalan T, Sahebkar A. Effect of resveratrol on C-reactive protein: An updated meta-analysis of randomized controlled trials. Phytother Res 2021;35(12):6754-67. doi: 10.1002/ptr.7262.

4. Koushki M, Dashatan NA, Meshkani R. Effect of Resveratrol Supplementation on Inflammatory Markers: A Systematic Review and Meta-analysis of Randomized Controlled Trials. Clin Ther 2018;40(7):1180-92 e5. doi: 10.1016/j.clinthera.2018.05.015.

5. Zeraattalab-Motlagh S, Jayedi A, Shab-Bidar S. The effects of resveratrol supplementation in patients with type 2 diabetes, metabolic syndrome, and nonalcoholic fatty liver disease: an umbrella review of meta-analyses of randomized controlled trials. Am J Clin Nutr 2021;114(5):1675-85. doi: 10.1093/ajcn/nqab250.

6. Koushki M, Lakzaei M, Khodabandehloo H, Hosseini H, Meshkani R, Panahi G. Therapeutic effect of resveratrol supplementation on oxidative stress: a systematic review and meta-analysis of randomised controlled trials. Postgrad Med J 2020;96(1134):197-205. doi: 10.1136/postgradmedj-2019-136415.
